# Gaps in care for patients with memory deficits after stroke: views of healthcare providers

**DOI:** 10.1186/s12913-017-2569-5

**Published:** 2017-09-08

**Authors:** Eugene Yee Hing Tang, Christopher Price, Blossom Christa Maree Stephan, Louise Robinson, Catherine Exley

**Affiliations:** 1 Institute of Health and Society and Newcastle University Institute for Ageing, Newcastle University, Level 2, Biomedical Research Building, Campus for Ageing and Vitality, Newcastle upon Tyne, NE4 5PL UK; 2 Institute of Neuroscience, Stroke Research Group, Newcastle University, Newcastle upon Tyne, UK; 3 Faculty of Health & Life Sciences, Northumbria University, Newcastle upon Tyne, UK

**Keywords:** Stroke, Memory, Cognition, General practitioners

## Abstract

**Background:**

Stroke is a common cause of physical disability but is also strongly associated with cognitive impairment and a risk for future dementia. Despite national clinical guidelines, the service provided for stroke survivors with cognitive and memory difficulties varies across localities. This study critically evaluated the views of healthcare professionals about barriers and facilitators to their care.

**Methods:**

Seventeen semi-structured individual interviews were conducted by a single interviewer with both primary and secondary care clinicians in regular contact with stroke-survivors. This included stroke medicine specialists, specialist nurses, physiotherapists, occupational therapists, general practitioners and primary care nurses. Topics included individual experiences of the current care offered to patients with cognitive impairment, assessment processes and inter-professional communication. Interviews were audio recorded and transcribed verbatim. Transcripts were thematically analysed and themes grouped into broad categories to facilitate interpretation.

**Results:**

Data analysis identified four key themes as barriers to optimal care for stroke-survivors with memory difficulties: 1) Less focus on memory and cognition in post-stroke care; 2) Difficulties bringing up memory and cognitive problems post-stroke; 3) Lack of clarity in current services; and, 4) Assumptions made by healthcare professionals introducing gaps in care. Facilitators included stronger links between primary and secondary care in addition to information provision at all stages of care.

**Conclusions:**

The care provided by stroke services is dominated by physical impairments. Clinicians are unsure who should take responsibility for follow-up of patients with cognitive problems. This is made even more difficult by the lack of experience in assessment and stigma surrounding potential diagnoses associated with these deficits. Service development should focus on increased cohesiveness between hospital and community care to create a clear care pathway for post-stroke cognitive impairment.

## Background

Stroke is a leading cause of morbidity worldwide and the third most common cause of disability [1]. This is not only a result of effects on motor function but because stroke is also associated with cognitive impairment and an increased risk of dementia [2–4]. Indeed, one in three people will experience stroke, dementia or both at some stage in their lives [5, 6]. After stroke around one in three individuals will sustain some degree of cognitive impairment [7] and memory deficits are commonly encountered even when physical recovery is gained [8]. Further, 10 % of individuals develop dementia soon after their first stroke and at least 30 % have dementia after recurrent stroke [3]. These cognitive deficits are not necessarily dictated by the severity of stroke and can also occur in transient or minor strokes [9].

Stroke care in the United Kingdom has been shaped by the National Service Framework for Older People (2001) [10] and the National Stroke strategy (2007) [11] although these do not specifically propose service organisation for patients with cognitive impairment. The Intercollegiate Stroke Working party recommends a collaborative model between primary and secondary care for long-term follow-up of those presenting with neuropsychological problems [12]. The Sentinel Stroke National Audit Programme (SSNAP) monitors whether assessment of cognition is included during routine six-month review prior to secondary care discharge, but the nature of the assessment and care pathway is not mandated. General Practitioner’s (GPs) are then traditionally tasked with ongoing management of secondary prevention post-stroke to try to minimise future risk of recurrent stroke [12].

It should be noted that understanding and reducing cognitive impairment was voted the single most important topic in stroke research in a national priority setting exercise [13, 14]; thus highlighting the importance to patients. Despite this level of prioritisation and the formal organisation of stroke services, gaps in care exist for patients with cognitive and memory difficulties. The National Audit Office’s report of stroke care highlighted the widespread lack of psychological services, which was also rated as the least satisfactory service in long-term care by patients [15]. Similarly, the Care Quality Commission reported that services need to address stroke-related cognitive problems [16] as only 40% of primary care trusts had good access to psychological therapy [16]. The first annual SSNAP report found that 4 in 10 people needing support for mood or memory after discharge did not get it [17]. A recent survey was carried out by the Stroke Association on 1424 stroke survivors across England detailing their own personal experiences of stroke care [18]. They found that 77% of stroke survivors have problems with memory and nearly 50% of stroke survivors reported poor support for mental fatigue and memory [18]. Although there is a focus to improve recognition of these problems by secondary care, many of these stroke-survivors may not present with cognitive deficits before discharge from specialist services. It is unclear what happens to these at-risk individuals when they are in the community. However, stroke survivors often report feeling abandoned when they leave hospital, which perhaps places emphasis on better community care and addressing psychosocial domains during rehabilitation on a par with physical recovery [18].

To improve the current model of care for stroke-survivors with cognitive and memory difficulties, it is first necessary to understand the barriers and facilitators from both the primary and secondary care perspective. This qualitative study used semi-structured interviews to describe primary and secondary care professionals’ views of care received by stroke-survivors with memory/cognitive difficulties.

## Methods

### Sampling

An initial list of primary (General Practitioners) and secondary care clinicians (Stroke consultants and specialist nurses) in the North East of England were contacted to participate in the study. Sampling for the study was purposive and iterative to identify additional participants from the relevant specialty that might have a unique perspective. Potential participants were approached by email to take part in the study. The email included a summary of the research and gave the opportunity to ask further questions. All participants approached agreed to further contact.

### Interviews

Face to face or telephone semi-structured interviews were conducted with both primary and secondary care staff (see Table 1). Interviews were conducted with 17 primary and secondary care clinicians who would be involved in the care of patients after their stroke at different stages of care. The interviews were conducted between May 2016 and February 2017 by one researcher (EYHT). The researcher (EYHT) utilised a topic guide, which evolved to ensure that emerging themes were explored. A topic guide provides a list of broad questions or areas to be covered during the interview. By responding to the data that emerged from these interviews, the topic guide evolved to address additional areas raised by participants themselves. Topics included the experience of clinicians in looking after stroke patients with memory difficulties, barriers and challenges to optimal clinical care and views on future care with emphasis on assessment for future dementia diagnoses. Participants were given the opportunity to discuss other issues they deemed important in the care of stroke-survivors with memory/cognitive difficulties. Informed written consent was obtained at the time of the interview including agreement for the interview to be audio-recorded. The interviews were transcribed verbatim. Unique identifiers were used throughout the process and any other identifiable information was removed to protect the anonymity of the participants.

**Table 1:** Details of included participants

Unique Identifier	Role
NSC1	Stroke Consultant
NSC2	Stroke Specialist Nurse
NSC3	Stroke Consultant
NSC4	Stroke Consultant
NSC5	Stroke Specialist Nurse
NSC6	Stroke Physiotherapist (Rehabilitation)
NSC7	Stroke Physiotherapist (Acute Care)
NSC8	Stroke Occupational Therapist (Acute Care)
NSC9	Stroke Occupational Therapist (Rehabilitation)
PC1	General Practitioner with Specialist Interest in Dementia
PC2	General Practitioner
PC3	General Practitioner
PC4	Nurse Practitioner
PC5	General Practitioner
PC6	Practice Nurse
PC7	Nurse Practitioner
PC8	General Practitioner

### Analysis

Data collection and analysis followed the principles of a thematic analysis [19]. One researcher (EYHT) familiarised himself with the data by repeated reading of the transcripts. Initial line-by-line coding was performed on the first few transcripts. A small subset of transcripts was read and subjected to coding and discussion between CE and EYHT to identify initial themes from the data. A coding framework was then developed between CE and EYHT through the application of thematic analysis [19] and codes were changed iteratively. Further analysis led to the generation of new themes and subsequent reviewing and refining of existing themes and subthemes. These themes and subthemes were subsequently grouped into broad categories to facilitate interpretation. The coding was facilitated by using a data software handling package (NVivo version 11).

## Results

### Barriers in current care of stroke-survivors with cognitive/memory difficulties

#### Less focus on memory and cognition in post-stroke care

The primary and secondary care participants repeatedly reported that post-stroke care often focussed exclusively upon the physical impact of the stroke. Although memory and cognition is not at the forefront of rehabilitation, its effects are evident as one participant remarked:

*“With some patients you see that that doesn’t actually sink in. Then you’re three weeks into rehab and you’ve seen no change, so it can really impact on the success of the interventions that we give. Sometimes there are patients that we can’t make any difference, and that’s quite hard for the patient – and for therapist, as well.” (NSC9, Occupational Therapist)*

Even upon discharge into the community, there is often less emphasis on communicating deficits in cognition and memory which was recognised by one primary care participant:

*“… Memory is very rarely mentioned in the discharge letter. It’s not something that is commonly mentioned, so it’s not a case of, “Well, this person has had a stroke, we would recommend that this person has the MMSE [Mini-Mental State Examination] checked every six months.” There is none of that; it’s more to do with, “His speech is better, he’s mobilising better, these are the tablets he’s on, can you check his kidney function in the week?” It’s that kind of thing.” (PC1, GP with Specialist Interest in Dementia)*

This may well be because the focus of care for clinicians is in improving the patient’s physical functioning to enable them to return home as soon as possible. This has meant that memory and cognition has not always been at the forefront of their training and practice:

*“I think that the focus in our training, be it medical, be it nursing, be it therapy, tends to be on physical impairment, so I don’t think we’ve got a good training base in it … Actually, it’s a blind spot in many services, and not just in terms of the service as a whole, but sometimes in how we are looking at helping people who’ve had a stroke, I think clinicians can have a very big blind spot to cognitive problems.” (NSC3, Stroke Consultant)*

However, it may well be that cognitive difficulties are not well prioritised by patients themselves and often become an after-thought rather than a main priority:

*“At that appointment they're often worried about the speech systems or the arm weakness or the leg weakness that they had and then it's sort of, “Any other problems?” and they sort of go, “Well, the memory's not so good.” It sort of comes up that way rather than it being a massive issue. So almost something that’s the whole hand on the door as they’re going out, ‘can we just tell you their memory’s not as good as it was?’ sort of thing.” (NSC1, Stroke Consultant)*

In the community, stroke-survivors may even accept this as part of their post-stroke recovery rather than seek to rectify the issue as noticed by some professionals:

*“A gentleman I saw recently who was … particularly in word-finding difficulty, but also some mild memory problems that he very much just lived with, and was put to one side by his family. It wasn’t their primary concern, they just said, “This is how things have been since the stroke,” and that was the end.” (PC8, GP)*

There is a feeling for clinicians that patients themselves may underplay their symptoms but that this may be driven by their perception that there is little to be done for their symptoms:

*“I think people might just consider it as a decline, as a general decline after the stroke, so it’s something that they might have envisaged anyway, something perhaps that they think, A, isn’t too serious, and B, well no-one can do anything about this anyway.” (PC5, GP)*

#### Difficulties bringing up cognition and memory problems post-stroke

In the context of stroke, both primary and secondary care professionals recognised memory and cognition to be difficult areas to raise with patients. In secondary care this would be because of the perceived additive negative effect another potentially life-changing diagnosis could have:

*“You’ve got to pick your moment, and when they come in here they get gloom and doom. The, “You’re not likely to survive.” The thing with a stroke as you know, it’s so sudden, you get no warning for it so there’s no psychological preparation for it, and it’s all pretty emergency, and people aren’t really taking it in. To give them another potentially devastating diagnosis I think would be quite difficult.” (NSC7, Stroke Physiotherapist)*

In primary care, experience of broaching this difficult topic was similar. However, this was more about the effect it may have on patients particularly when they have recovered from their stroke physically but still had ongoing cognitive issues.

*“Sometimes, I think, as a nurse, it’s quite difficult to sit down with somebody who’s doing fantastically well after their stroke, to say, “Actually, how’s your memory?” It’s just another whole thing. You’re trying to be on a positive note saying. “You’re doing really well.” I think sometimes it’s just difficult to actually bite the bullet and say, “How is your memory?” I think it doesn’t get broached very well.” (PC7, Primary Care Nurse Practitioner)*

Indeed, one participant commented that undoubtedly, some individuals would rather be seen to have a physical limitation than a cognitive one:

*“I think they'd rather have a physical disability that can be dealt with than something that's invisible, but definitely, it's impacting on their life and the whole family's lives.” (PC6, Practice Nurse)*

There may be gaps, either in structure or communication between primary and secondary care teams. Once patients have been deemed safe for discharge, according to professionals, the responsibility of care and follow-up for these deficits is then given back to the patients or at least that is what secondary care professionals expect upon discharge from the stroke service:

*“I think, a lot of the time, we, you know, I tend to put the onus back on the patient and the relatives, say, for example. And say to them, that if they feel that it’s starting to become a problem, then they should go and see their GP sooner rather than later.” (NSC5, Stroke Specialist Nurse)*

However, professionals suggested that patients themselves may not wish to bring up their memory problems, mask their symptoms or even make excuses as to why things have changed so suddenly without acknowledging the potential underlying problem. This can be found in secondary care but then persist into the community:

*“Or sometimes trying to cover up that they’re having these problems, as well, wanting to appear like everything’s okay.” (NSC6, Stroke Physiotherapist)*

*“I think, partly, it’s a bit of, sort of, maybe some denial that,” “Well, I’m getting a bit forgetful, but don’t we all get like that, especially since I’ve been poorly?” (PC2, GP)*

Although some participants found that this may be because patients would prefer to minimise these symptoms, there were other reasons noted by clinicians. Clinicians felt that some patients had challenges communicating these symptoms:

*“Not always, no, because I don't think they know how to verbalise it. So they would probably... It depends on the patient and the conversation you're having, but some of them might make a joke about it or it might be the spouse that brings it up and then you can sort of investigate it and question it and drill down a little bit more. But I don't think they really... I don't think they know how to say.” (PC4, Nurse Practitioner)*

#### Lack of clarity in current services

Both primary and secondary care clinicians felt that the current service pathway was inadequate to ensure optimal management of stroke-survivors with cognitive difficulties. Time in consultation was consistently felt to be a significant barrier at all stages of post-stroke care:

*“I think - It’s a difficult one, because I think the way we are set up in the NHS [National Health Service], both in secondary and primary care, we don’t necessarily have the time to probably care for these people how we need to.” (PC2, GP)*

Participants felt that this may mean that patients are not given sufficient support or indeed the relevant information regarding the sudden post-stroke changes they are facing:

*“I just think that some patients and families in particular might need a bit more help to understand the impact that that aspect of the stroke has had upon the patient, and how that is impacting on their relationships, their communications, and different changes.” I don’t think that we necessarily spend as much time as we should on helping people understand the change, and what that means, and how that can be helped, or how that can be dealt with. It’s often just taken as read, “This has happened, get on with it.” (NSC3, Stroke Consultant)*

A key area of concern was the lack of, or inconsistent level of, social care support for these individuals:

*“Social care has got to be funded to care for these people. These people don't want to be in residential care, necessarily. They don't want to be blocking beds in hospitals. They want to be in their homes, amongst their surroundings, where it's familiar, but they need help to be able to do that. They've got to pump more money into the social care, and train up people specifically to look after these people.” (PC6, Practice Nurse)*

Several participants were also unsure where the care of patients should take place but recognised the limitations it would have in either setting. The current pathway of care mainly involves primary care taking over once individuals are discharged from stroke services. However, GPs reported being more reactive in the care of these patients. GPs would often watch and wait for symptoms to become more evident rather than undertaking any formal risk assessment:

*“I'm not even sure how I would record that in the records, to indicate that there had been some potential impairment picked up, but no action currently required. I think I would probably be fairly reliant on that just becoming evident over time, that further assessment was required or more needed to be done. Gosh, that feels quite uncomfortable saying that, actually.” (PC3, GP)*

#### Assumptions made by healthcare professionals introducing gaps in care

It was well recognised by both primary and secondary care clinicians that there are gaps in care, particularly for stroke-survivors who go on to develop cognitive difficulties. These gaps could lead to unmet needs:

*“I think there is a big number of patients who certainly have got ongoing needs that perhaps aren’t having them fully addressed” (NSC8, Stroke Occupational Therapist).*

Gaps in care may exist because of assumptions made by both primary and secondary care participants. Participants from primary and secondary care would often comment on what they perceived to be happening for stroke-survivors upon discharge from stroke services. Secondary care clinicians saw their roles as bringing together the information and then expect GPs to refer these individuals:

*“We’re [stroke clinicians] basically summarising the issues, and usually there’s an expectation, unless it’s very gross, that primary care will pick it up ... I think what we would probably be doing, actually, is if it’s causing enough concern to the family, and the patients, we would be at that stage probably expect GPs to refer (them) into the local memory clinic service” (NSC4, Stroke Consultant)*

However, according to primary care health professionals, there was an assumption that secondary care had perhaps investigated and found that no further action was required. This lack of action may well be because stroke services do not have the capacity to take on longer term cognitive issues and so redirect to the community. Primary care professionals suggested this might have the inadvertent consequence of implying to patients that GPs are disinterested because no further action is taken despite the fact that these patients still have ongoing issues:

*“Well, the patient has been asked a question in a secondary care setting, and have answered that honestly, in that yes they perhaps have noticed a change in their memory. Secondary care have explored, found that there’s no further action required at that time, and the patient has been discharged back to us. We’ve received a letter saying those things. Then we appear disinterested, potentially, and the patient’s perception… That’s imagining that I'm seeing it from the patient’s point of view. “Well, the doctors at the hospital couldn’t do anything. The GP and the doctor has taken no further action. Nobody cares. Nobody is interested. Nobody wants to do anything.” Maybe how it’s interpreted, yes, now I’m thinking about it that way round. That’s what makes me feel uncomfortable” (PC3, GP)*

### Facilitators to improve the current care of stroke-survivors with cognitive/memory difficulties

As well as barriers to care, participants were also asked about how they could improve the current care pathway. This fell into two broad categories as outlined below:

## Stronger links between primary and secondary care

Links between primary and secondary care structures and staff were felt to be important to many participants. Indeed, one participant, when asked on how to ensure gaps in care were filled talked about the need for clarity in the care pathway:

*“We need to be clear on when that patient actually needs to be seen again rather than leaving it as an open”, “We think they’re at risk, will you see them? Will you bear this in mind?” It needs to be kind of a clear pathway of saying, “Well actually this needs to be reviewed again by a certain date.” (NSC2, Stroke Specialist Nurse).*

To ensure this could be put in place, one participant expanded on how best to achieve this clarity. This included clear communication between the two systems:

*“I think it would be useful if hospital discharge letters do mention, if there are any issues with memory, if those problems are actually mentioned on the hospital discharge letter, I think that definitely would be quite useful”. Or even if the discharge letter said something like, “During assessment his MMSE score was 26 out of 30; although we’re not too worried about it, I would be grateful if you could repeat it in six months.” (PC1, GP with specialist interest in dementia)*

Besides better communication between the two teams, participants suggested that the whole team (primary and secondary care) needed to take ownership in delivering this care:

*“I don’t think anybody should take sole ownership of it. I think it’s up to everybody, and that’s where a good MDT [multidisciplinary team] works well in a hospital. Here it works that we all do our own jobs, but we all do a little bit of everybody else’s because we work very closely, so you’re picking up different things. In the community, it really depends who’s involved in the ongoing care. So I think everybody should have an awareness of it. It shouldn’t just be one person, but then there should be some sort of pathway to follow to make sure that these patients are being given the care or the information that they need.” (NSC7, Stroke Physiotherapist)*

However, it was also recognised that patients should have the choice whether to access a relevant service rather than automatic enrolment onto a cognitive post-stroke pathway:

*“I believe that we should have specialist stroke services available, preferably in partnership with primary care, where there is a structured follow-up available for people who want it, and where there is open access to people who don’t want it, who just want to have access then.” (NSC3, Stroke Consultant)*

## Information provision at all stages of care

The interview data suggest that information about post-stroke memory problems is not always provided in the first place or presented in a digestible format for the patient. Participants felt that it was important that the patient and their families are equipped to manage their cognitive deficits. This means that patients need to be identified as having a need and then given and taught the skills to ensure their safety in the community:

*“But you need to be giving people the skills to be able to manage those risks and be able to live to whatever quality of life is possible, in a safe manner, without having to have constant health professional support. So I think it’s about having that support, but also teaching skills so that people don’t need that support all the time, so that you can increase their self-efficacy with dealing with their cognitive problems.” (NSC6, Stroke Physiotherapist)*

Participants also suggested that it is important for clinicians not to be fearful of disclosing more information, particularly if the patient and their families are keen to explore further. This may well involve charitable organisations but requires the clinician to be proactive to look out for opportunities to do so:

*“Part of the role of the NHS professionals is to signpost appropriately, and maybe offer information about organisations like Alzheimer’s Society. Or possibly even have a sort of direct conduit in. So there could be a formal referral at that point, if the patient and/or their carers felt that they would benefit from some support, from whoever is doing the feedback on the results. “It doesn’t look as though there’s another explanation for this memory impairment. In all likelihood it is a consequence of the stroke. However, there is an organisation who would be willing to offer some support. Would you like me to ask them to make contact with you?” That would feel like an ideal way.” (PC3, GP)*

Finally, professionals felt that accessible information about cognitive trajectories post-stroke and when to seek help were fundamental if patients held the responsibility:

*“If you notice somebody that’s at risk or you notice somebody that’s developed these things, you monitor and track it over time. You then have the opportunity to tell the family what kind of things to look for, what kind of things they should be prepared for, and then maybe have a chance to refer to the right people, give them the right numbers.” (NSC9, Stroke Occupational Therapist)*

## Discussion

This study has reported clinicians’ accounts of some barriers, which they have encountered when looking after stroke-survivors with subsequent memory problems. Four key barriers were identified including: 1) Less focus on memory and cognition in post-stroke care; 2) Difficulties bringing up cognition and memory problems post-stroke; 3) Lack of clarity in current services; and, 4) Assumptions made by healthcare professionals introducing gaps in care. The relationships between these factors are also important to consider. For example, although rehabilitation of physical disabilities and a short length of stay may be the reason why there is less focus on memory post-stroke, this is compounded by the difficulties in starting conversations with patients about their cognitive difficulties. The lack of clarity in service provision means that it becomes even more difficult to ensure optimal care. Clinician participants here recognised that there is indeed a gap in the care for these individuals and have highlighted some areas which could be improved upon: 1) Stronger links between primary and secondary care; and, 2) Information provision at all stages.

It is not just clinicians who place less focus on cognitive and memory issues. In the context of stroke, the focus of rehabilitation generally is on physical recovery, as this tends to be the dominant symptom post-stroke. Indeed, one participant remarked that patients would rather have an impairment that could be seen than one such as cognition, which is not so obvious. Similarly, in a small sample of stroke patients, the patients themselves also failed to include cognitive deficits in their perception of overall recovery with the focus almost solely on the physical impairments [20]. This is despite the fact it was recognised later on that their memory loss deficits had negatively influenced their daily functional activities [20]. If clinicians struggle to discuss the issue of memory or cognitive loss and patient’s themselves place less priority on these symptoms, identification will become increasingly challenging. The presence of cognitive impairment post-stroke has important functional consequences which are independent from the effects the physical impairments encountered post-stroke [21]. Emphasising the need to focus on both cognitive and physical impairments is necessary to ensure stroke survivors continue to function well in the community. This is currently a challenge, as access to psychological support is limited [22]. There are recommendations to ensure that patients’ access to psychological support are as important as their access to physical support services [22] particularly for those where cognitive dysfunction only becomes apparent when they are living in the community.

Stroke patients and their caregivers require information if they are to seek help appropriately particularly with regards to cognitive and memory impairment. Patients themselves report either dissatisfaction with or a lack of information provision following a stroke [23]. Amongst health professionals this may well be because individuals are unaware which professional is providing the information [24]. Even when provided, recall of information for those with memory difficulties post-stroke poses a significant challenge for the patient and their carer [25]. Although it is assumed that stroke clinicians provide the required information post-stroke, the GP takes over this role in the community. However, evidence has shown that patients often receive the majority of their information from stroke services rather than primary care [26]. The highest risk of post-stroke dementia seems to occur in the first months post-stroke, although this may be partially due to pre-stroke cognitive impairment [27]. However a population based study with 25 year follow-up found that the cumulative incidence of post-stroke dementia was 7% at 1 year, 23% at 10 years and 48% at year 25 [28]. A further study found looked at cognition post-stroke over time. They found that although 41% were stable and 50% improved in cognition after 15 months, a significant proportion of post-stroke survivors succumbed to delayed post-stroke dementia [29, 30]. This suggests that stroke-survivors need to have adequate follow-up in the community and continual access to information and support to ensure prompt and timely diagnosis of post-stroke dementia. Stronger links between specialist and community teams could help identify those at-risk and assist in targeted cognitive assessment and follow-up.

Professionals in this study commented on the difficulties of broaching the subject of memory impairment post-stroke. When participants in this study considered the patient’s perspective, they commented on patients often masking, normalising or denying the existence of their memory loss symptoms. In the context of cognitive deficit, masking or denial of symptoms is often attributed to a barrier in the earlier diagnosis of dementia [31]. The patient’s reluctance to begin conversations regarding memory concerns may well be due to an underlying fear of developing another significant life-changing diagnosis. From the clinicians’ point of view, concerns about additional burden or indeed stigmatisation of patients with a diagnosis of dementia [32] and unwillingness to discuss cognitive problems with patients or caregivers [31, 33] is not unique to post-stroke care. In general a significant proportion of people with dementia remain undiagnosed [34] with groups such as those living alone, men and the oldest old may be at particular risk of missed diagnosis [35]. The additive effect of another significant symptom, particularly when stroke-survivors have recovered from their physical impairment, may contribute towards this barrier. Future work will need to explore patients’ views in more detail particularly with regards to barriers in disclosing cognitive difficulties following their stroke.

The strength of this study is that we have been able to capture the views of the majority of healthcare professionals who would encounter stroke-survivors with memory or cognitive deficits post-stroke. The spectrum of views has included those in acute post-stroke care and their subsequent care in the community. There are some limitations to this study. This was a qualitative piece of research conducted in one area of England. The results may therefore not completely capture other practices nationally. However, the care pathway for stroke patients is governed by national policies to ensure a minimal standard of care and it is likely that these views are representative of other settings. In future, the experience and views resulting from alternative and international models of care may further add to our understanding of how we can improve patient care. Finally, views from patients and carers would certainly provide a vital perspective about the impact of gaps in care. We are currently undertaking data collection with these groups.

## Conclusions

Cognitive and memory impairment post-stroke is common and can significantly hinder daily functioning. Health professionals involved in the care of stroke patients commented upon barriers to care, which are evident along the whole patient pathway. As recommended by experts in the field [12], there should be a focus on improvements in the coordination and cohesiveness of hospital and community care in support of stroke patients who have or are at risk of developing cognitive problems.

## Abbreviations

GP: General Practitioner
MMSE: Mini-Mental State Examination
NHS: National Health Service
SSNAP: Sentinel Stroke National Audit Programme
